# Evaluation of the automated MicroFlow^®^ and Metafer™ platforms for high-throughput micronucleus scoring and dose response analysis in human lymphoblastoid TK6 cells

**DOI:** 10.1007/s00204-016-1903-8

**Published:** 2016-12-10

**Authors:** Jatin R. Verma, Benjamin J. Rees, Eleanor C. Wilde, Catherine A. Thornton, Gareth J.S. Jenkins, Shareen H. Doak, George E. Johnson

**Affiliations:** Institute of Life Science, School of Medicine, Swansea University, Swansea, SA2 8PP UK

**Keywords:** Micronucleus, Automation, Dose response, Metafer™, MicroFlow^®^

## Abstract

The use of manual microscopy for the scoring of chromosome damage in the in vitro micronucleus assay is often associated with user subjectivity. This level of subjectivity can be reduced by using automated platforms, which have added value of faster with high-throughput and multi-endpoint capabilities. However, there is a need to assess the reproducibility and sensitivity of these automated platforms compared with the gold standard of the manual scoring. The automated flow cytometry-based MicroFlow^®^ and image analysis-based Metafer™ were used for dose response analyses in human lymphoblastoid TK6 cells exposed to the model clastogen, methyl methanesulfonate (MMS), aneugen, carbendazim, and the weak genotoxic carcinogen, ochratoxin A (OTA). Cells were treated for 4 or 30 h, with a 26- or 0-h recovery. Flow cytometry scoring parameters and the Metafer™ image classifier were investigated, to assess any potential differences in the micronucleus (MN) dose responses. Dose response data were assessed using the benchmark dose approach with chemical and scoring system set as covariate to assess reproducibility between endpoints. A clear increase in MN frequency was observed using the MicroFlow^®^ approach on TK6 cells treated for 30 h with MMS, carbendazim and OTA. The MicroFlow^®^-based MN frequencies were comparable to those derived by using the Metafer™ and manual scoring platforms. However, there was a potential overscoring of MN with the MicroFlow^®^ due to the cell lysis step and an underscoring with the Metafer™ system based on current image classifier settings. The findings clearly demonstrate that the MicroFlow^®^ and Metafer™ MN scoring platforms are powerful tools for automated high-throughput MN scoring and dose response analysis.

**Electronic supplementary material:**

The online version of this article (doi:10.1007/s00204-016-1903-8) contains supplementary material, which is available to authorized users.

## Introduction

The in vitro micronucleus assay is a robust platform for the assessment of chromosomal damage following the treatment of genotoxic agents. In this assay, a quantitative measure of the induced chromosomal damage (chromosomal breaks and chromosomal loss) is acquired by scoring micronuclei (MN) (Fenech 2000). These events can be detected following mitosis, where the lost or broken chromosome resides in the cytoplasm, and not the nucleus. Traditionally, MN scoring is carried out manually by using bright field or fluorescent microscopy. However, the manual scoring procedure has been scrutinised for its subjectivity and extensive scoring time (Doherty et al. 2011; Seager et al. 2014).

To overcome these issues, efforts have been made to automate the MN scoring platform. These include the use of both the semi-automated and the fully automated MN scoring approaches that are compatible with multi-endpoint MN analysis and high-through scoring (Bryce et al. 2007; Varga et al. 2004). Commercially available platforms such as the Litron Laboratories automated flow cytometric platform (MicroFlow^®^) and the semi-automated image analysis platform (Metafer™ and Pathfinder™) are among the most widely used MN scoring procedures. The Metafer™ MN scoring platform is often used in the pharmaceutical industry and in academia to assess the genotoxic potential of various DNA damaging agents, and it shows a good concordance with conventional MN scoring platform (Chapman et al. 2014).

The MicroFlow^®^ MN scoring platform is proposed as a viable alternative to the manual scoring to conduct objective, multi-parametric MN scoring, with reduced data acquisition time using flow cytometry. Furthermore, the incorporation of nuclear stains ethidium monoazide (EMA) allows discrimination of apoptotic bodies and necrotic cells from MN which can be difficult to define manually, and re-probing with pan nuclear stain SYTOX green following cell lysis provides precision MN scoring (Avlasevich et al. 2006). Even so, it is likely that chromatin from a certain fraction of early-stage apoptotic cells may not always be excluded from analysis based on EMA staining. Also, cells with multiple MN and multi-nucleated cells with MN would be scored differently from lysed (nuclei) preparations compared with intact cells. We predict that both of these situations would tend to result in somewhat higher flow cytometry-based MN frequencies relative to microscopy.

The aim of the present study was to assess the reproducibility of the MN dose responses generated with the MicroFlow^®^ and Metafer™ systems as compared to traditional manual scoring. For this purpose, human lymphoblastoid TK6 cells were treated with a clastogen (MMS), an aneugen (carbendazim) and a DNA damaging agent (ochratoxin A), with the cells scored using the three different approaches.

## Methods and materials

### Chemicals

Methyl methanesulfonate (CAS no. 12925), carbendazim (CAS no. 10605-21-7) and ochratoxin A (CAS no. 303-479) were purchased from Sigma-Aldrich, UK.

### Cell lines and treatment

Human lymphoblastoid TK6 cells were obtained from American Type Culture Collection (ATCC), Manassas, VA, USA. TK6 cells were cultured in RPMI 1640 media (Gibco, Paisley, UK), supplemented with 1% pen-strep and 10% heat inactivated horse serum (Gibco, Paisley, UK). Cells were seeded at 2 × 10^5^ cells in 25-cm^2^ flask (Fisherbrand), incubated at 37 °C for either 4 or 30 h (1.5–2 cell cycles) in the presence of MMS, carbendazim and ochratoxin A (OTA). Subsequently, the treatment was removed and the cells were harvested following 0- or 26-h recovery period. Resulting MN was scored in the absence of cyto-B by using the Metafer™ (MetaSystems, Althlussheim, Germany) and the MicroFlow^®^ (Litron laboratories, Rochester, USA) platforms. The manual scoring procedure was used as a validation tool to verify the results between the MicroFlow^®^ and the Metafer™ scoring procedures.

### Cytotoxicity and cytostasis

Cell counts were determined using a Coulter counter (Beckman Coulter Inc.). Relative population doubling (RPD) was used to estimate the highest cytotoxic concentration. MN scoring was restricted to the concentration that induced 50% cell death and cytostasis. The RPD calculation is described in detail elsewhere (Lorge et al. 2008)

$$\% {\text{RPD}}\;{ = }\;\frac{{{\text{Number}}\;{\text{of}}\;{\text{population}}\;{\text{doubling}}\;{\text{in}}\;{\text{treated}}\;{\text{cultures}}}}{{{\text{Number}}\;{\text{of}}\;{\text{population}}\;{\text{doubling}}\;{\text{in}}\;{\text{the}}\;{\text{vehicle}}\;{\text{control}}}}\; \times 100$$

Population doubling (PD) was calculated as follows:

$${\text{PD }} = {\text{ Log }}\left( {{\text{Cell count after treatment}}/{\text{cell count in the control}}} \right)/{ \log }2.$$

### The manual scoring procedure

Cells were harvested following 4- or 30-h treatment. Briefly, treated cells were transferred to 15-ml centrifuge tubes and were centrifuged at 200×*g* for 10 min. Supernatant was aspirated, and the pellet was re-suspended in 10 ml phosphate-buffered saline (Gibco^®^). Subsequently, the cell suspension was cytospun (Cytospin™ centrifuge) on a polished glass slides, fixed in 90% ice cold methanol for 10 min and were air-dried at room temperature.

Air-dried slides were stained in 4% Giemsa solution (VWR International Ltd., Poole, UK) at room temperature. Giemsa stained slides were washed under tap water and air-dried, and a cover slip was mounted on these slides using DPX mounting solution. Mononucleated cells with intact nuclear and cytoplasmic membrane were considered suitable for MN identification. The parameters used for MN scoring were size (between 1/3rd and 1/16th the diameter of nuclei), morphology (circular or oval) and their association with the main nuclei (not linked or overlapping the nuclei) (Fenech et al. 2003). The MN scoring was carried out by using 20× magnifications on a light microscope (Olympus BX 51). The MN frequency was obtained by manually assessing 2000 mononucleated cells per replicate. A total of 6000 mononucleated cells were scored using the manual scoring platform.

### Metafer™ analysis

Cells were harvested post-treatment. At the time of harvest, treated cells were transferred to 15-ml centrifuge tubes (Fisherbrand) and centrifuged at 200×*g* for 10 min. Supernatant was aspirated, and the pellet was re-suspended in hypotonic solution 5% KCl (KCL, 75 Mm; Sigma-Aldrich). The cell suspension was centrifuged, supernatant was removed, and the pellets were fixed in 5 ml of Fix 1 [methanol/acetic acid/NaCl (5:1:6)] for 10 min at room temperature. Fix2 (methanol/acetic acid 5:1, Fisher Scientific) was used to re-suspend the pellet following centrifugation. Cells were incubated in Fixative 2 for 10 min at room temperature and centrifuged at 4 °C, 200×*g* for 10 min. These pellets were re-suspended in Fixative 2 and stored overnight at 4 °C.

For Metafer™ analysis, 100 μl of cell suspension was dropped on to a polished glass slide. Slides were then air-dried, and 20 µl of 4,6-diamidino-2-phenylindole (Vector Laboratories, Peterborough, UK) was use to label nuclei and MN. A cover slip was mounted, and slides were incubated for 15 min at room temperature. Subsequently, the MN induction was assessed using a semi-automated Metafer™ MN scoring platform (Meta System, Althlussheim, Germany). The Metafer MN scoring platform consists of a motorised slide loading platform, Carl Zeiss Axio Imager fluorescence microscope and a charge-coupled device (CCD) camera. Image acquisition was carried out by using Metafer 4 software (version 3.9.8).

Stained slides were loaded on to a motorised slide scanning platform of Metafer system. Slides were scanned; images of nuclei and MN were captured with 10× objective. A 100× objective was used for MN scoring by relocating the cell and MN on the slide form the coordinates displayed in the gallery view. Non-overlapping, DAPI stained circular/oval nuclei with a size between 1/3rd and 1/16th of the main nuclei were scored as MN (Fenech et al. 2003). A total of 18,000 mononucleated cells were assessed to enumerate MN frequency.

### The MicroFlow^®^ approach

Total 5 × 10^5^ treated cells were transferred to 15-ml centrifuge tube and were centrifuged at 300×*g* for 5 min. The supernatant was aspirated, and the pellets were incubated on ice for 20 min. The cells were stained with ethidium monoazide (EMA) following 30-min photo-activation. During this incubation period, the cells were placed on ice 2 cm below the source of light. This process was used to label cell with compromised cytoplasmic membrane. The fold change in EMA-positive events was used alongside %RPD to estimate increased cytotoxicity and to predict highest test concentration. A greater than fourfold increase in EMA-positive event was used as an indicator of increased apoptosis/necrosis (Bryce et al. 2013). The cytoplasmic membrane and the cellular RNA were digested by using detergents and RNase solution following photo-activation step. Subsequently, the nuclei and MN were labelled with SYTOX Green stain. Stained samples were then incubated overnight prior to flow cytometric analysis.

### Flow cytometric scoring

Prior to the flow cytometric assessments, the suspension of sequentially stained nuclei and MN was incubated at room temperature for 30 min. Samples were acquired on a flow cytometer (BDFACS Aria, BD Biosciences, USA) equipped with 488-nm laser, and BD FACS Diva software (version 6.1.3) was used for MN scoring. EMA-associated fluorescence collected in the FL3 channel was used to monitor increased levels of apoptotic/necrosis. Scoring of nuclei and MN was limited to the cells that displayed SYTOX-associated fluorescence signals in FL1 channel. With the MicroFlow^®^ approach, the viable mononucleated cells were detected from their SYTOX Green-associated fluorescence, DNA content as determined by side scatter and size based on the forward scatter characteristics. For an event to be classified as MN with the MicroFlow^®^ approach, the MN should not be labelled with EMA, exhibit SYTOX Green fluorescence between 1/10th and 1/100th for the main nuclei and should fall in the side and forward scatter regions (Bryce et al. 2007). A total of 24,000 events that displayed SYTOX intensities were used to enumerate MN frequency.

### Statistical analysis

Shapiro–Wilk normality test, Bartlett test or homogeneity of variance and Bonferroni test for outlier identification were conducted. Data were transformed in order to achieve normally distributed data and homogeneity of within-dose variance. If the raw or transformed data passed these trend tests, then the 1-sided Dunnett’s test was used to identify the no-observed and the lowest observed genotoxic effect levels (NOGEL, LOGEL) and if the data failed these trend tests, then the 1-sided Dunnett’s test was used (Johnson et al. 2014).

Covariate benchmark dose (BMD) analysis was carried out using PROAST (v60.12) to compare dose responses (Slob 2002). This approach relies on constant shape parameters for log-steepness and maximum response being used between each independent dose response, which provides increased precision for each dose response and allows for potency ranking to be carried out (Soeteman-Hernández et al. 2016; Wills et al. 2016a, b). In this instance, it was carried out to observe any trends in equipotency or not between the chemicals and MN scoring approach. Overlapping BMDs show that equipotency cannot be rejected and non-overlapping BMDs show that there is a difference. Furthermore, when there is no response at the concentrations tested, conserved shape information from the other responses is used to fit suitable models to allow for BMDL to be derived but with infinite BMDU.

## Results

### Cytotoxicity and cytostasis

The 50 ± 5% reduction in percentage RPD is a standardised method to estimate highest test concentration for accurate MN enumeration (OECD 2014). The fold change in EMA-positive events alongside percentage RPD was used to monitor apoptosis/necrosis at the highest test concentration.

**Fig. 1:**
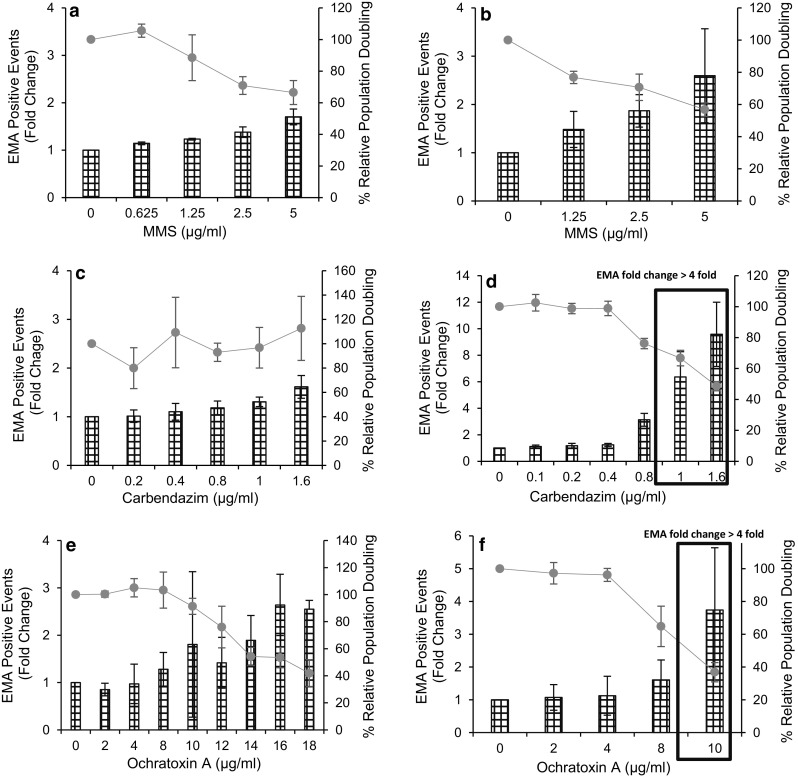
Cytotoxic and apoptotic/necrotic effects of MMS (**a**, **b**), carbendazim (**c**, **d**) and OTA (**e**, **f**) in TK6 cells following 4-h (*left-hand panel*) or 30-h (*right-hand panel*) treatment. The mean percentage RPD (*blue solid lines*) and EMA-positive fold change (histograms) were used as parameters to assess cytotoxicity (*n* = 3). Overly cytotoxic concentration (*black box*) as indicated by  %RPD or fold change in EMA-positive events (≥4 fold increase above the control) or both (Bryce et al. 2013) (colour figure online)

The concentration of 5 μg/ml MMS was selected as the highest test concentration to cause 50 ± 5% cytotoxicity, following a 4- or 30-h treatment (Fig. 1a, b). At this test concentration, no evidence of increased cytotoxicity and cytostasis was seen from the %RPD and the fold change in EMA-positive events. In response to 5 μg/ml MMS, the %RPD dropped to 66% following 4 h and 56% following 30-h treatment. The fold change in EMA-positive events, a 1.7-fold increase following 4-h treatment and a 2.5-fold increase following a 30-h treatment in response to 5 μg/ml MMS, was well below the cut-off (≥4-fold) change for a dose to be considered overly cytotoxic.

Carbendazim did not cause any increase in cytotoxicity or apoptosis/necrosis in TK6 cells following 4-h treatment (Fig. 1c). In contrast, increased apoptosis/necrosis was evident for the fold change values for EMA-positive events following 30-h continuous treatment. Sixfold and 9.5-fold increases in EMA-positive events were observed for 1 and 1.6 μg/ml concentrations. These fold change values for EMA staining were greater than fourfold increase above the control for these concentrations and hence considered overly cytotoxic.

Contradictory results were also seen in TK6 cells following 4-h treatment with OTA. The 18 μg/ml concentration of OTA was identified as overly cytotoxic as 41% RPD (59% cytotoxicity) was seen at this dose (Fig. 1e). In contrast to %RPD, a 2.5-fold increase in EMA-positive fold change was recorded at the same analysed concentration. Following 30-h continuos treatment, 10 μg/ml OTA was identified as overly cytotoxic by both %RPD and fold change in EMA-positive events (See Fig. 1f). Therefore, MN enumeration was limited to 8 μg/ml concentration of OTA following continuous treatment.

### Evaluation of MN induction using the automated MN scoring platforms

**Fig. 2:**
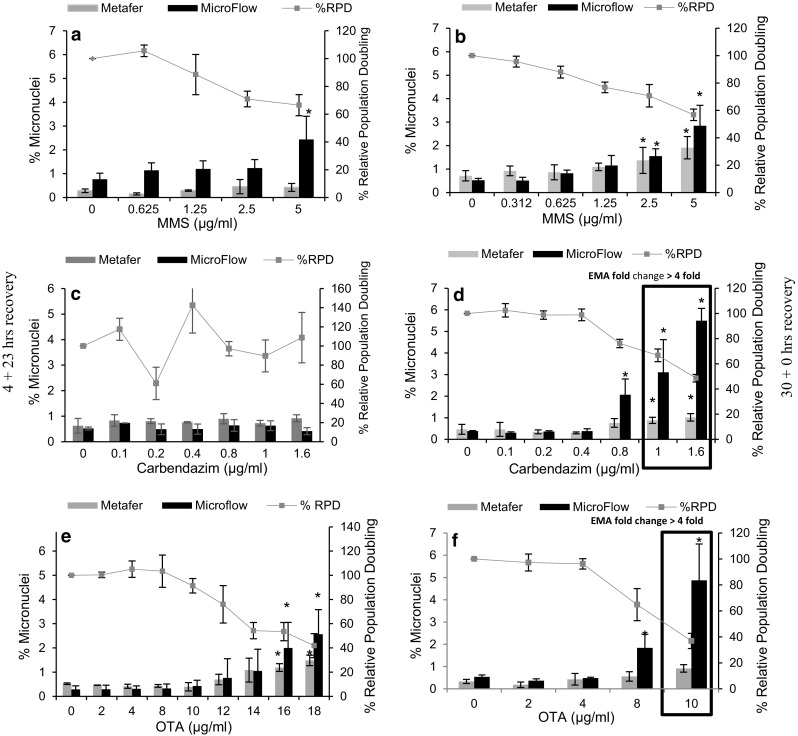
Genotoxic effects of MMS, carbendazim and OTA in TK6 cells following 4-h (*left-hand panel*) and 30-h (*right-hand panel*) treatment. The mean MN frequencies derived by the MicroFlow (*black bars*) approach and the Metafer (*grey bars*) scoring platforms. Increased cytotoxicity (*black box*) as indicated by  %RPD and fold change in EMA-positive events (≥4-fold increase above the control). *Asterisk* indicates a significant increase in the MN formation over the control using a (*p* < 0.05). *Error bars* represent mean ± SD (*n* = 3). The  %RPD values in these graphs are same as those seen in Fig. 1

In the case of MMS, discrepancies were seen between the MN dose responses when using the Metafer and the MicroFlow approaches, following 4-h treatment (Fig. 2a). The Metafer scoring platform did not detect any significant increase in the MN induction following 4-h treatment. In contrast, a significant increase in MN frequency was detected at 5 μg/ml MMS when scoring was carried out using the MicroFlow approach. The mean MN responses were comparable between the scoring platforms in TK6 cells treated continuously for 30 h (Fig. 2b). Both the systems detected a significant (*p* < 0.05) increase in MN induction in response to 2.5 and 5 μg/ml MMS.

In the cells treated with carbendazim, no increase in MN frequency was detected following 4-h treatment by either platform. Using the MicroFlow approach, a significant increase in MN was observed at 0.8, 1 and 1.6 μg/ml carbendazim at 30 h. However, increased apoptosis/necrosis was also seen when measuring fold change in the EMA-positive events at 1 and 1.6 μg/ml concentrations. The Metafer MN scoring platform detected a significant (*p* < 0.05) increase in MN frequencies at carbendazim concentrations of 1 and 1.6 μg/ml.

OTA induced a significant (*p* < 0.05) increase in the MN induction above control at 16 and 18 μg/ml was detected by both the MN scoring platforms following 4-h treatment (Fig. 2c). In contrast, conflicting results were seen following 30-h continuous treatment with OTA (Fig. 2d). In this instance, no increase in the MN frequency was detected when using the Metafer platform at the analysed test concentrations, whereas a clear increase in MN induction above the control was seen at 8 μg/ml OTA with the MicroFlow approach.

Furthermore, the MN frequencies obtained following 30-h treatment of MMS, carbendazim and OTA were comparable to those obtained in the cytokinesis block micronucleus assay using Metafer platform (please see supplementary data, Fig. 6)

### The manual scoring approach

Significant differences were seen between the MN responses derived by using the MicroFlow^®^ and the Metafer™ scoring platforms in TK6 cells following a 4-h treatment of MMS and 30-h OTA. To resolve this issue, the manual scoring procedure was used alongside the MicroFlow^®^ and the Metafer™ scoring platforms to assess MN induction at 4 h using MMS and carbendazim.

**Fig. 3:**
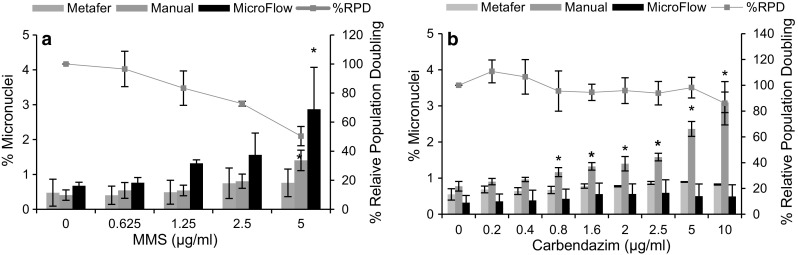
Comparison of the MN responses derived by the MicroFlow (*black bars*), manual scoring (*green*) and the Metafer (*grey bars*) in TK6 cells treated with MMS and carbendazim for 4 h. *Asterisk* indicates a significant increase in the MN formation over the control using a (*p* < 0.05). *Error bars* represent mean ± SD (*n* = 3) (colour figure online)

In the case of MMS, MN dose response derived following 4-h MSS treatment by using the manual scoring method was comparable to that of the MicroFlow^®^ approach (Fig. 3a). Both the MicroFlow^®^ and manual scoring approaches detected a significant (*p* < 0.05) increase in MN frequency at 5 μg/ml MMS concentration. In contrast, no significant increase in MN induction was seen when the scoring was carried out using the Metafer™ platform.

Surprisingly, the MN response derived in TK6 cells with the manual scoring platform following 4-h treatment of carbendazim was different to those obtained using the MicroFlow^®^ and the Metafer™ scoring platforms (Fig. 3b). In this instance, a significant increase in MN induction was observed when manual scoring at 0.8 μg/ml and concentrations above it. In contrast, no such increase in the MN formation was seen when using the MicroFlow and the Metafer scoring platforms.

### Covariate BMD analysis

**Fig. 4:**
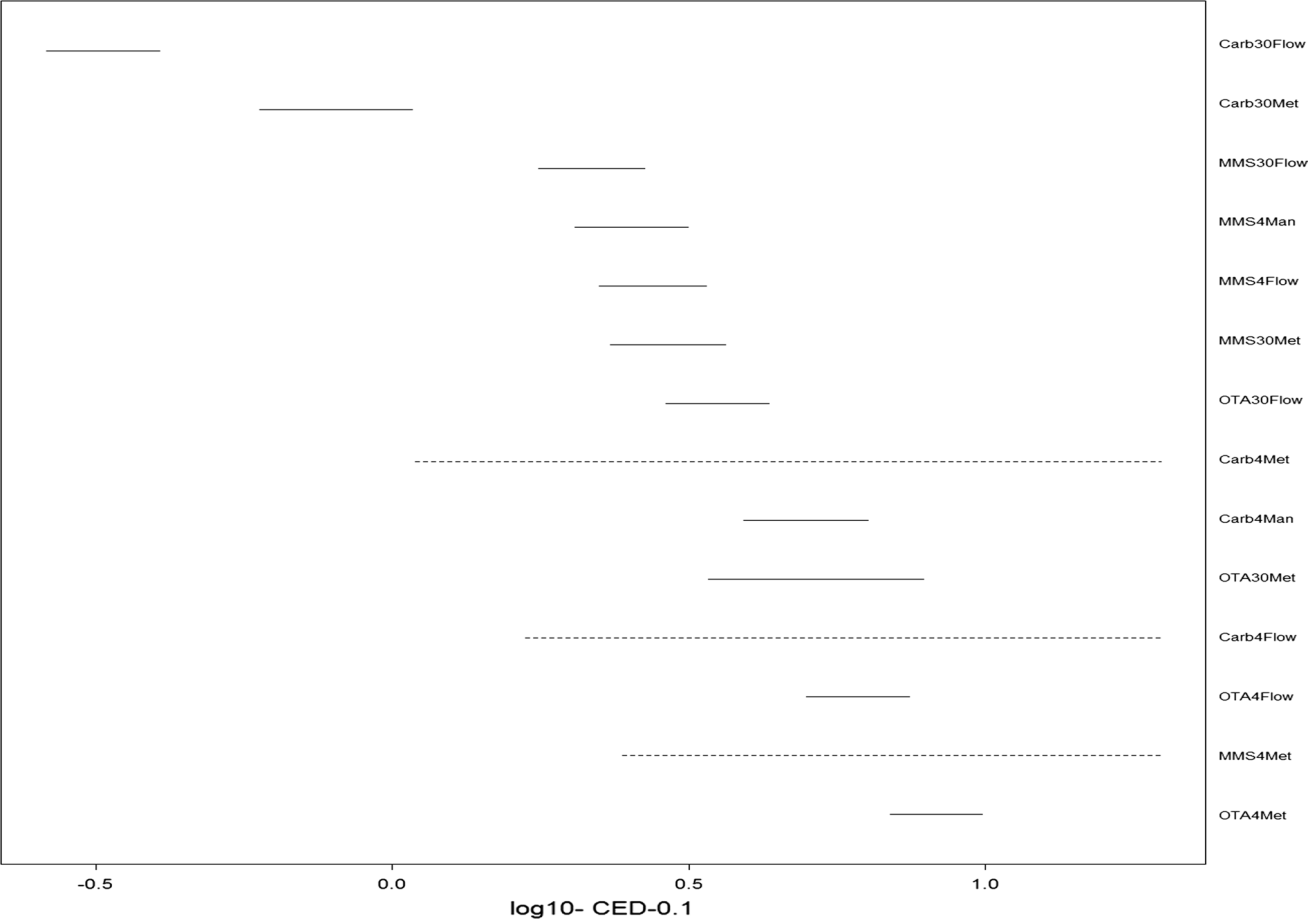
MN BMD Covariate analysis, potency ranking, from most potent/sensitive *top left* to least potent/sensitive bottom right. X-axis, Log10.dose (μg/ml). Carb, carbendazim; 30, 30-h treatment; 4, 4-h treatments, flow, MicroFlow; met, Metafer; man, manuals scoring

The order of endpoint sensitivity was deduced from the covariate BMD analysis, where the horizontal lines represent the BMDL_10_-BMDU_10_ metrics, with the lines in the top left being the lowest and most sensitive, and the ones on the bottom right being the highest and therefore least sensitive. Overlapping lines show equipotency between endpoints, and dotted lines represent poor model fits with infinite BMDL_10_ metrics (Fig. 4). The potency evaluations show that the MicroFlow approach was the most sensitive when compared to other techniques at 30 h. Using Metafer at 30-h chemical exposure was used to accurately characterise MN for all three chemicals, but for OTA it did produce wider BMD confidence intervals than the other approaches. Metafer at 4-h exposure was not suitable for MMS or carbendazim, but was suitable for the assessment of OTA.

For MMS, the BMD confidence intervals were indistinguishable between 30-h MicroFlow, 4-h manual, 4-h MicroFlow and 30-h Metafer with high precision, whereas 4-h Metafer provided the least precision in the BMD estimate. For carbendazim at 30-h treatment, the lowest BMD metrics were provided by MicroFlow followed by Metafer. At 4-h time-point, both Metafer and manual provided equivalent metrics, but the MicroFlow did not provide a good estimate of the BMD. Following 30-h treatment, MicroFlow and Metafer provided equivalent BMD metrics. At 4 h, MicroFlow and Metafer provided equivalent BMD metrics that were non-distinguishable from 30-h Metafer due to the wide confidence intervals, but with higher BMDs than for 30-h MicroFlow.

### Carbendazim altered the morphology of the micro nucleated cells and induced larger MN

The greatest discrepancies among scoring platforms were seen for TK6 cells treated with carbendazim. Whilst scoring MN using the manual scoring platform, it was observed that the nucleus of these micronucleated cells was crescent-/kidney-bean-shaped. Thus, it was speculated that these nuclear anomalies alongside large size MN were causing misclassification of micronucleated cells and MN with the Metafer scoring platform. The Metafer uses predefined parameters such as the size, aspect ratio, eccentricity and DAPI staining intensity for the detection of nuclei and MN (Varga et al. 2004). Therefore, any deviations from these parameters for observed nuclei/MN will have a significant effect on MN frequency, and such MN cells will be excluded resulting into lower proportion of micronucleated cells. Studies with spindle poisons have previously shown to induce larger MN in TK6 and NH32 cells (Hashimoto et al. 2012). Hence, it was postulated that carbendazim-induced MN were larger and thus not appropriately identified by Metafer classifier that had been standardised on micronucleated cells induced by clastogens. Hence, further MN scoring in TK6 cells treated with Carbendazim was carried out manually by using florescent microscopy in cells stained with DAPI and chromosomes counter stained with human pan centromeric probes. With this dual staining approach, two parameters such as the morphology of the micronucleated cells and the number of centromeric signals with the MN were evaluated to address the issue of underscoring with the Metafer system. A total of 100 micronucleated cells were assed for the occurrence of larger MN (MN with 2 or more centromeric signals) and morphologically altered MN cells.

**Fig. 5:**
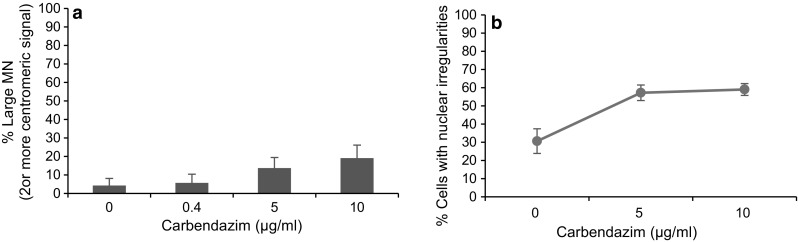
FISH to assess the induction of larger MN (**a**) and assessment of morphologically altered MN cells (**b**) in TK6 cells treated with carbendazim

Carbendazim caused a concentration-dependent increase in the number of micronucleated cells with morphologically abnormal nuclei (Fig. 5a) and large size MN (Fig. 5b) in TK6 cells treated for 4 h. These results clearly indicate that the classifier standardised for detecting MN induced by clastogens might not be suitable to detect MN induced by aneugens.

## Discussion

The reproducibility, sensitivity and transferability of the MicroFlow^®^ and Metafer™ approaches were compared with manual scoring through analyses of the dose response data. Using this approach, the MicroFlow^®^ data were comparable to the Metafer™ data for MMS, carbendazim and OTA, although there was a clear difference in the MN response magnitude. This difference could be due to underscoring by Metafer™ current classifier settings, where cells with novel nuclear morphology are not identified, or where there is misclassification of large MN as nuclei. However, this could be overcome with a visual detection step (Decordier et al. 2009), or an updated classifier. A potential for overestimation of the MN frequencies with the MicroFlow^®^ approach could be due to the cell lysis step, where MN is not always associated with a single mononuclear cell. In both cases, the fold change in EMA-positive events along side %RPD was considered suitable to estimate cytotoxic concentration and to study apoptosis/necrosis.

The flow-based MN scoring procedure provides benefits over manual scoring and, to an extent, semi-automated Metafer in terms of high-throughput MN scoring and multiplexing. With the MicroFlow approach, 10,000 events (cells) can be scored within a minute, whereas it takes up to 3 min to visually certify images of MN derived from 3000 thousands cells with Metafer and 15 min to visually inspect 1000 cells with the manual scoring platform. In addition to automated MN scoring, the MicroFlow approach permits assessment of additional cellular parameters such as cell cycle changes and apoptosis/necrosis which are otherwise difficult to assess using the conventional platforms. The BMD covariate analysis showed that for MMS and OTA, the Metafer and MicroFlow approaches produced equivalent BMD metrics, but for the aneugen carbendazim, the MicroFlow provided the most sensitive BMD estimates which were achieved at 30-h treatment. However, at 4 h, neither the Metafer or MicroFlow approaches were suitable for deriving BMD metrics.

One major disadvantage of using the MicroFlow MN approach is the inability to differentiate bi-, tri- and multi-nucleated cells with MN and cells with multiple MN. This can lead to elevated MN frequencies compared with analyses conducted with intact cells. This effect was seen clearly in response to MMS and Carbendazim (Fig. 2). Doherty et al. (2014) also observed an increase in the frequency of micronucleated bi-nucleated cells with MMS in non-cyto-B treated cells (Doherty et al. 2014). Additionally, the origin of the MN via clastogenic or aneugenic mechanisms cannot be elucidated following cell lysis procedure with the MicroFlow approach, although some cell lines (e.g., CHO-K1) are reported to provide aneugenicity signatures that include hypodiploidy and increased median MN fluorescence intensity (Bryce et al. 2010).

There are some issues in using the MicroFlow^®^ approach, such as re-validation of the misleading positive and negative result following cell lysis and flow cytometric analysis and stained samples cannot be store for a long period when compared with Metafer and manual scoring where slide can be store for months (Fenech et al. 2013). However, there are also some disadvantages when using the Metafer system, with the major one being that some MN events are not picked, which leads to the underscoring as shown in the dose responses for all the three chemicals (Fig. 2). Since the samples were prepared from the same treated culture it was postulated that the Metafer™ classifier settings were incompatible for scoring MN induced by the aneugen Carbendazim. With the Metafer™ platform, the classifier is configured to assess parameters such as shape, circularity, aspect ratio and size to detect nuclei and MN (Reference). Therefore, it is possible that subtle changes in the morphology of nuclei/MN and induction of larger MN could cause underscoring with this system. The FISH and morphological studies provided some evidence on the induction of larger MN and morphologically altered nuclei in TK6 cells exposed to Carbendazim (Fig. 5a, b), which is in line with previous studies (Hashimoto et al. 2010). However, a larger sample size and increase doses are required to confirm these findings for carbendazim, and this hypothesis also needs to be tested on other aneugens. The advantages and disadvantages of the each of these different scoring methodologies for scoring MN are summarised in Table 1. In order for these approaches to be more widely used for MN scoring and dose response analysis, future ring trials should focus on addressing these considerations as well as assessing interlaboratory reproducibility.

**Table 1:** Summary of the advantages and disadvantages of manual, Metafer™ and the MicroFlow^®^ approaches

MN scoring approaches	Scoring platforms	Advantages	Disadvantages
Image analysis	Manual microscopy (light microscopy)	Suitable for dose response and mode of action analysis Simple, economical and adaptable Suitable for MN scoring in the presence or the absence of cyto-B Stained slides can be stored for a long time and can be re-analysed Suitable for assessing bi-, tri- and poly-nucleated cells	Interoperational variations can result in subjective MN scoring Slow, tedious and time-consuming Lack multiplexing abilities Total number of cells scored manually is limited which reduces the overall statistical power
	Metafer™ (fluorescent microscopy)	Semi-automated platform High content for higher statistical precision Suitable for dose response and mode of action analysis for most substances Images of nuclei and MN can be stored for re-validation	Classifier settings have to be optimised for different cell lines and chemicals that induce MN via varied mechanisms Lack of cytoplasmic staining, detection of small MN and manual validation of the images
Flow cytometry	MicroFlow^®^	Fully automated platform to score MN objectively Suitable for dose response High content and high throughput Permits cell cycle analysis 10,000 events scored in 1–2 min	Cell lysis is required prior to MN scoring Misleading MN cannot be re-validated from same sample Overestimation and underestimation of MN are both possible and require expert analysis Lack of MOA analysis with TK6 cells

Both the MicroFlow and Metafer scoring approaches are suitable for automated MN scoring. However, in cases of equivocal with chemicals with unknown activity, it may be advisable to additionally process the same treated samples for manual scoring. These manually scored slides can be used to reduce the occurrence of misleading results, assess cytotoxicity or conduct mechanistic studies. Whilst conducting MN scoring on the semi-automated Metafer system, the classifier setting should be adopted to account for chemical or cell line-specific morphological changes and to reduce the occurrence of misleading results (positive and negative). These semi-automated and fully automated platforms can therefore be used for dose response analysis as substantially higher number of cells can be scored with these methods which allows for much statistical power.

A test system that combines the high high-throughput, high-content and multiplexing potential of flow cytometry, with the re-validation and data storage benefits for image analysis, would be a major step forward in achieving a truly twenty-first century approach.

## Electronic supplementary material

Below is the link to the electronic supplementary material.

Supplementary material 1 (PPTX 1078 kb)

## References

[CR1] Avlasevich SL, Bryce SM, Cairns SE, Dertinger SD (2006). In vitro micronucleus scoring by flow cytometry: differential staining of micronuclei versus apoptotic and necrotic chromatin enhances assay reliability. Environ Mol Mutagen.

[CR2] Bryce SM, Bemis JC, Avlasevich SL, Dertinger SD (2007). In vitro micronucleus assay scored by flow cytometry provides a comprehensive evaluation of cytogenetic damage and cytotoxicity. Mutat Res.

[CR3] Bryce SM, Shi J, Nicolette J, Diehl M, Sonders P, Avlasevich S, Raja S, Bemis JC, Dertinger SD (2010). High content flow cytometric micronucleus scoring method is applicable to attachment cell lines. Environ Mol Mutagen.

[CR4] Bryce SM, Avlasevich SL, Bemis JC, Tate M, Walmsley RM, Saad F, Van Dijck K, De Boeck M, Van Goethem F, Lukamowicz-Rajska M, Elhajouji A, Dertinger SD (2013). Flow cytometric 96-well microplate-based in vitro micronucleus assay with human TK6 cells: protocol optimization and transferability assessment. Environ Mol Mutagen.

[CR5] Chapman KE, Thomas AD, Wills JW, Pfuhler S, Doak SH, Jenkins GJ (2014). Automation and validation of micronucleus detection in the 3D EpiDerm human reconstructed skin assay and correlation with 2D dose responses. Mutagenesis.

[CR6] Decordier I, Papine A, Plas G, Roesems S, Vande Loock K, Moreno-Palomo J, Cemeli E, Anderson D, Fucic A, Marcos R, Soussaline F, Kirsch-Volders M (2009). Automated image analysis of cytokinesis-blocked micronuclei: an adapted protocol and a validated scoring procedure for biomonitoring. Mutagenesis.

[CR7] Doherty AT, Hayes J, Fellows M, Kirk S, O’Donovan M (2011). A rapid, semi-automated method for scoring micronuclei in mononucleated mouse lymphoma cells. Mutat Res/Genet Toxicol Environ Mutagen.

[CR8] Doherty AT, Wilson A, Chocian K, Molloy J, Clatworthy M, Doak S, Jenkins G, O’Donovan M (2014). Genotoxins induce binucleation in L5178Y and TK6 cells. Mutat Res, Genet Toxicol Environ Mutagen.

[CR9] Fenech M (2000). The in vitro micronucleus technique. Mutat Res.

[CR10] Fenech M, Chang WP, Kirsch-Volders M, Holland N, Bonassi S, Zeiger E (2003). HUMN project: detailed description of the scoring criteria for the cytokinesis-block micronucleus assay using isolated human lymphocyte cultures. Mutat Res.

[CR11] Fenech M, Kirsch-Volders M, Rossnerova A, Sram R, Romm H, Bolognesi C, Ramakumar A, Soussaline F, Schunck C, Elhajouji A, Anwar W, Bonassi S (2013). HUMN project initiative and review of validation, quality control and prospects for further development of automated micronucleus assays using image cytometry systems. Int J Hyg Environ Health.

[CR12] Hashimoto K, Nakajima Y, Matsumura S, Chatani F (2010). An in vitro micronucleus assay with size-classified micronucleus counting to discriminate aneugens from clastogens. Toxicol In Vitro.

[CR13] Hashimoto K, Nakajima Y, Uematsu R, Chatani F (2012). Difference in susceptibility to morphological changes in the nucleus to aneugens between p53-competent and p53-abrogated lymphoblastoid cell lines (TK6 and NH32 cells) in the in vitro micronucleus assay. Mutagenesis.

[CR14] Johnson GE, Soeteman-Hernandez LG, Gollapudi BB, Bodger OG, Dearfield KL, Heflich RH, Hixon JG, Lovell DP, MacGregor JT, Pottenger LH, Thompson CM, Abraham L, Thybaud V, Tanir JY, Zeiger E, van Benthem J, White PA (2014). Derivation of point of departure (PoD) estimates in genetic toxicology studies and their potential applications in risk assessment. Environ Mol Mutagen.

[CR15] Lorge E, Hayashi M, Albertini S, Kirkland D (2008). Comparison of different methods for an accurate assessment of cytotoxicity in the in vitro micronucleus test. I. Theoretical aspects. Mutat Res.

[CR16] OECD (2014). OECD TG 487: in vitro mammalian cell micronucleus test.

[CR17] Seager AL, Shah UK, Brusehafer K, Wills J, Manshian B, Chapman KE, Thomas AD, Scott AD, Doherty AT, Doak SH, Johnson GE, Jenkins GJ (2014). Recommendations, evaluation and validation of a semi-automated, fluorescent-based scoring protocol for micronucleus testing in human cells. Mutagenesis.

[CR18] Slob W (2002). Dose-response modeling of continuous endpoints. Toxicol Sci.

[CR19] Soeteman-Hernández LG, Johnson GE, Slob W (2016). Estimating the carcinogenic potency of chemicals from the in vivo micronucleus test. Mutagenesis.

[CR20] Varga D, Johannes T, Jainta S, Schuster S, Schwarz-Boeger U, Kiechle M, Patino Garcia B, Vogel W (2004). An automated scoring procedure for the micronucleus test by image analysis. Mutagenesis.

[CR21] Wills JW, Johnson GE, Doak SH, Soeteman-Hernandez LG, Slob W, White PA (2016). Empirical analysis of BMD metrics in genetic toxicology part I: in vitro analyses to provide robust potency rankings and support MOA determinations. Mutagenesis.

[CR22] Wills JW, Long AS, Johnson GE, Bemis JC, Dertinger SD, Slob W, White PA (2016). Empirical analysis of BMD metrics in genetic toxicology part II: in vivo potency comparisons to promote reductions in the use of experimental animals for genetic toxicity assessment. Mutagenesis.

