# Supplementary material for: Evaluation of the automated MicroFlow® and Metafer™ platforms for high-throughput micronucleus scoring and dose response analysis in human lymphoblastoid TK6 cells
Source: Arch Toxicol. 2016 Dec 10;91(7):2689–98. doi: 10.1007/s00204-016-1903-8 (PMC5489585; doi:10.1007/s00204-016-1903-8)
Supplement: Supplementary file 1 — Supplementary material 1 (PPTX 1078 kb) [file 204_2016_1903_MOESM1_ESM.pptx]

## Slide 1
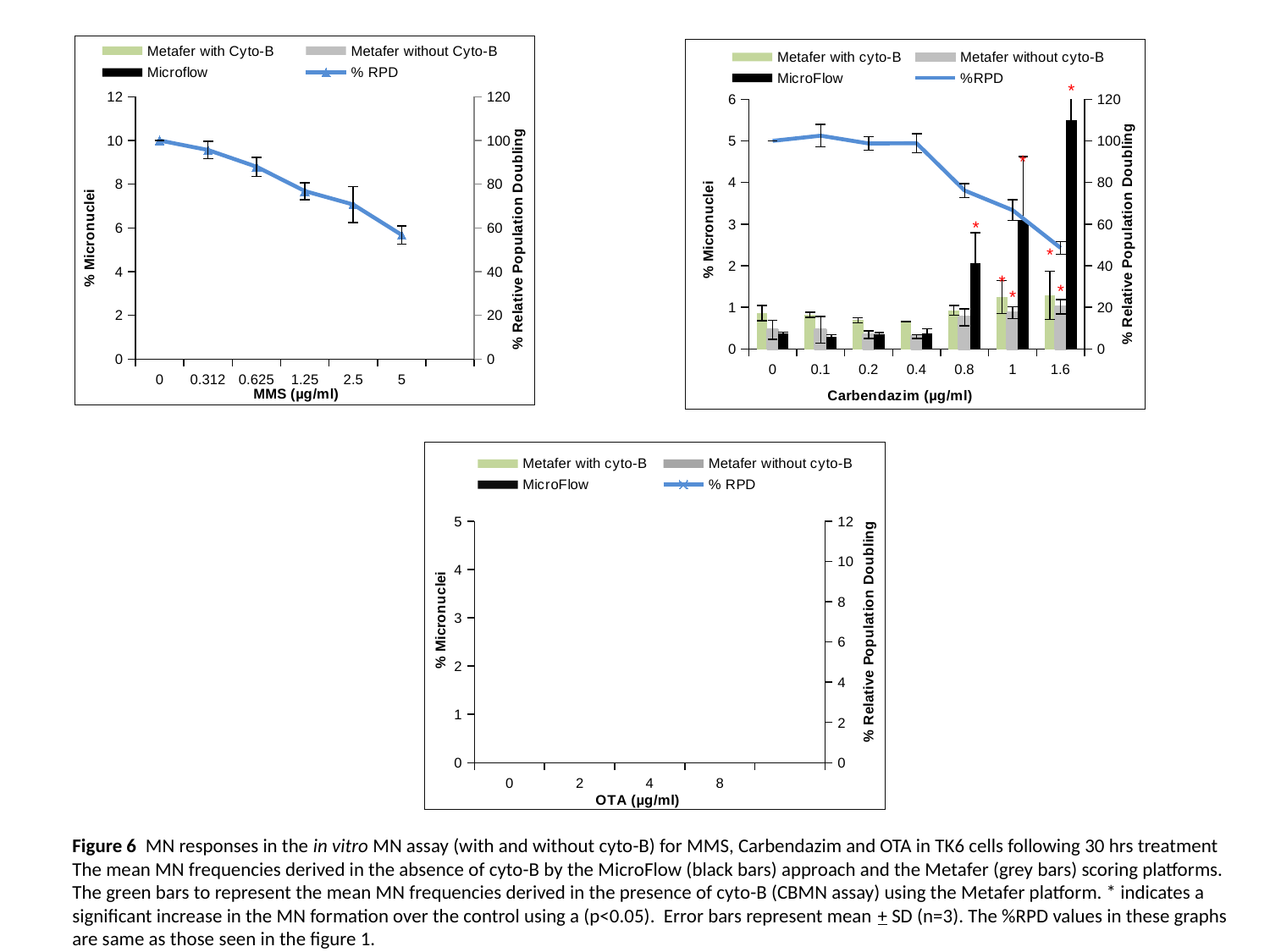

### Chart
| Category | | | | |
|---|---|---|---|---|
| 0 | 0.8868782322513137 | 0.7130694934456251 | 0.5228861556512762 | 100.0 |
| 0.312 | 1.0954350417899033 | 0.9249081983487988 | 0.5101436935426641 | 95.61347160271353 |
| 0.625 | 1.2277082842104559 | 0.862807213730239 | 0.819509748373196 | 87.96705548169002 |
| 1.25 | 1.314841486636011 | 1.0916181922929702 | 1.1558901747715156 | 76.82000303157636 |
| 2.5 | 1.63560941826406 | 1.3712053496296606 | 1.5513288712875457 | 70.69771726743974 |
| 5 | 2.0598241894944525 | 1.9109122052859355 | 2.8450367215511494 | 56.79628591481844 |
### Chart
| Category | | | | |
|---|---|---|---|---|
| | 0.8618054669313664 | 0.46233385733263854 | 0.37866598433656845 | 100.0 |
| | 0.8229441583873501 | 0.4601484263832354 | 0.3012243018949181 | 102.50197287510485 |
| | 0.6919447679446712 | 0.34607434103097795 | 0.3600659159467299 | 98.75545850425233 |
| | 0.6652332039007666 | 0.29717298080429105 | 0.37918216133384197 | 98.86856682309097 |
| | 0.9303229601101829 | 0.7601079884550068 | 2.065327502990507 | 76.16578705914917 |
| | 1.2482403667251905 | 0.877150082653171 | 3.1049037435059716 | 66.73055943117284 |
| | 1.2895480657006133 | 1.0155596575864443 | 5.496115520619647 | 48.569646279537494 |
### Chart
| Category | | | | |
|---|---|---|---|---|
| | 0.4582907489063508 | 0.3312023756316192 | 0.5371900636960711 | 100.0 |
| | 0.39173627350781004 | 0.1838922452273224 | 0.3639109041996254 | 97.23387032222806 |
| | 0.48832497233074407 | 0.4297464990116802 | 0.4833673383766787 | 96.22495077667817 |
| | 0.43478981915248854 | 0.5565828079327421 | 1.8390761783813352 | 64.82300791679283 |Figure 6 MN responses in the in vitro MN assay (with and without cyto-B) for MMS, Carbendazim and OTA in TK6 cells following 30 hrs treatment The mean MN frequencies derived in the absence of cyto-B by the MicroFlow (black bars) approach and the Metafer (grey bars) scoring platforms. The green bars to represent the mean MN frequencies derived in the presence of cyto-B (CBMN assay) using the Metafer platform. * indicates a significant increase in the MN formation over the control using a (p<0.05). Error bars represent mean + SD (n=3). The %RPD values in these graphs are same as those seen in the figure 1.
